# Perspectives of Muslim Religious Leaders to Shape an Educational Intervention About Family Planning in Rural Tanzania: A Qualitative Study

**DOI:** 10.9745/GHSP-D-22-00204

**Published:** 2023-02-28

**Authors:** Andrea Chalem, Aneth Nzali, Alexandra A. Cordeiro, Amina Yussuph, Evarist Laizer, Gregory Lupilya, Malick Lusana, Nelusigwe Mwakisole, Ndalloh Paul, Hidaya Yahaya, Abubakari Abdalah, Samuel E. Kalluvya, Valencia J. Lambert, David J. Downs, Albert Kihunrwa, Jennifer A. Downs, Agrey H. Mwakisole

**Affiliations:** a Center for Global Health, Weill Cornell Medicine, New York, NY, USA.; b Mwanza Christian College, Mwanza, Tanzania.; c Bugando Medical Centre, Mwanza, Tanzania.; d Misungwi District Hospital, Mwanza, Tanzania.; e Nyamagana District Hospital, Mwanza, Tanzania.; f Catholic University of Health and Allied Sciences, Mwanza, Tanzania.; g Keble College, University of Oxford, Oxford, United Kingdom.

## Abstract

**Introduction:**

Uptake of effective contraceptive methods can be hindered by poor understanding and uncertainty about its compatibility with religious beliefs. We sought to understand the perspectives of Muslim religious leaders in rural Tanzania on family planning (FP) and acceptable strategies for providing FP education to leaders and their communities.

**Methods:**

We conducted in-depth interviews with Muslim leaders from 4 communities in northwest Tanzania. Open-ended questions explored leaders’ views on FP in relation to their communities, Muslim texts and teaching, and their experience as leaders. We also investigated how FP education could be provided in their communities and asked practical questions regarding seminar implementation. Interviews were conducted in Kiswahili and transcribed and translated into English. Data were coded independently by 2 investigators using NVivo 1.5.1 and analyzed thematically.

**Results:**

We interviewed 17 male and 15 female Muslim leaders. All leaders supported FP as a concept in which births are spaced, interpreting this as espoused by the Qur’an and a basic right of children raised in Islam. Leaders uniformly endorsed the use of breastfeeding and the calendar method to space births but had divergent and sometimes opposing views on other methods, including condom use, oral contraceptives, and intrauterine devices. All leaders acknowledged the need for FP education among their congregants and were in favor of helping to teach an FP seminar in their communities.

**Conclusion:**

Our data reveal insights into how education for Muslim leaders may equip them to promote birth spacing and enhance understanding of FP in their communities in ways that are concordant with Islamic teaching. Our findings will guide the design and pilot-testing of an educational intervention for Muslim religious leaders to promote knowledge and uptake of FP in rural Tanzania.

## Abstract (precis)

Designing an educational intervention that engages male and female Muslim religious leaders and addresses gaps in knowledge on all contraceptive methods is a promising strategy for increasing family planning uptake in rural Tanzania.

## Teaser

**Key Findings:**

- Muslim religious and lay leaders in rural Tanzania interpreted Islamic teachings as accepting of family planning (FP) for birth spacing and uniformly supported spacing through breastfeeding and the calendar method.
- The need for more FP education in Muslim communities was widely agreed upon, and leaders were open to helping to provide this education.
- Leaders’ insights yielded recommendations for educating Muslim religious leaders in their communities. These included educating men and women both together and separately depending on context and partnerships between medical and religious leaders to co-teach educational sessions.
- Muslim religious leaders can be empowered to teach their own communities by receiving education about contraceptive methods, including how the methods work, their efficacy, and potential side effects.

**Key Implication:**

- Community education on FP in Muslim communities must engage male and female religious leaders. This study’s findings will guide the design and pilot-testing of an educational intervention for Muslim religious leaders to promote FP knowledge and uptake in rural Tanzania.

## INTRODUCTION

Spacing births by at least 24 months averts morbidity and mortality and is recommended by the World Health Organization to promote the health of mothers and infants worldwide.^1^ Implementation of family planning (FP) practices can significantly reduce maternal and infant mortality, poverty, and hunger^2^ and increase women’s access to education and opportunities for employment, ultimately improving their socioeconomic status.^3^^–^^9^ In 2008, it was estimated that FP saved the lives of 22,000 women across Ethiopia, Kenya, Rwanda, Tanzania, and Uganda, and that an additional 25% reduction could be achieved in maternal deaths per year in sub-Saharan Africa if current unmet needs for contraception are fulfilled.^10^ In data from Tanzania collected between 2004 and 2016, a short birth interval of less than 2 years and a birth order of fourth and higher were significant risk factors for neonatal, post-neonatal, and infant mortality.^11^

Unmet need for contraception often persists despite the fact that modern contraception is available free of charge in government clinics in many countries with high unmet need.^12^^–^^15^ We and others working in sub-Saharan Africa have previously demonstrated that religious beliefs can pose a major barrier to uptake of modern contraception, even among people who would like to delay childbearing.^16^^–^^19^ In particular, we recently documented uncertainty among Tanzanian Muslim men and women about whether modern contraception is compatible with their faith.^17^ Further, beliefs about contraception often vary even between congregations of the same religious sect or denomination and may depend highly on local teachings.^20^^,^^21^ Education on FP in the context of religious faith may be an effective way to address divergent views and increase uptake, particularly in rural communities where people tend to have less education and exposure to mass media.^22^ In rural Tanzania, an educational seminar designed to empower Christian leaders to educate their communities about FP has been pilot-tested with preliminary positive effects.^23^

Education on FP in the context of religious faith may be an effective way to address divergent views and increase uptake.

Less is known about whether and how local Muslim leaders could similarly be empowered to provide education about FP in their communities. An intervention in 20 districts in Pakistan that focused on educating health providers about the congruence of FP with Muslim faith was associated with increased uptake of modern contraception,^24^ but little work of this nature has been done in sub-Saharan Africa. In 2 Muslim communities in Kenya, Muslim leaders and scholars generally agreed that Islam supports nonpermanent contraceptive methods.^25^ Yet, despite leaders’ views, many Muslim believers in these and other communities stated that all contraceptives are *haram* (forbidden) and saw them as a Western concept that conflicts with the Islamic value of giving birth to as many children as possible and trusting Allah to provide.^25^^–^^27^

Given our prior data indicating that Muslim community members in Tanzania were unsure about the compatibility of FP with their faith, we sought to explore the perspectives of the Muslim leaders of these communities on FP and on the need for FP education in their communities. We conducted in-depth interviews with male Muslim leaders and female Muslim elders who were viewed as leaders in their communities in rural Tanzania. We further asked for these leaders’ opinions on best practices for designing and implementing an educational intervention about FP that would be acceptable and appropriate for equipping Muslim religious leaders to educate their communities about FP.

## METHODS

### Study Setting

We conducted in-depth interviews with Muslim leaders living in the Mwanza region of northwest Tanzania. We selected 4 communities, each of which had at least 1 mosque attended by 50 or more members, that were spread over an area of 450 km^2^. Leaders were purposively sampled by visiting each mosque and asking to speak with men and women over a range of ages who were either official or lay leaders at that mosque, as well as by snowball sampling through referrals of other Muslim leaders.

### Data Collection and Analysis

After participants gave their written informed consent, interviewers who had been trained in research ethics and qualitative research conducted in-depth interviews in a private setting in Kiswahili. Interviewers, who were from outside participants’ communities, interviewed participants of the same gender as their own to allow participants to speak more freely about gender and FP issues, which has been shown in our prior work.^17^ We used an interview guide with open-ended questions to explore how participants viewed FP in relation to their communities, Islamic texts and teaching, and their leadership roles (Supplement). We also sought participants’ views on practical aspects of teaching about FP in their communities. The interview guide was shaped by the team’s prior interviews with Christian leaders and was further developed and refined by all participating interviewers for appropriateness in Muslim communities. The team of interviewers held a daily debriefing session to discuss challenges encountered during interviews and ways to improve data collection the following day, as well as to assess when they perceived they were nearing data saturation.

Interviews were digitally recorded, transcribed verbatim in Kiswahili, and professionally translated into English. English transcripts were reviewed for accuracy against Kiswahili transcripts and clarified when needed by an investigator fluent in both English and Kiswahili. Transcripts were imported into NVivo 1.5.1 for subsequent coding.

We conducted a thematic analysis in which we systematically coded, compared, and sought to understand overarching themes in the data.^28^ After familiarizing themselves with the data, 2 investigators (AC and JAD) each reviewed 3 different transcripts and generated an initial list of codes. Initial codes were compared and revised to clarify meaning, presented to other study team members for additional input and refinement, and then used for coding of subsequent transcripts. Additional codes were generated in vivo when new themes were identified. Investigators met periodically during the coding process and at the completion of coding for peer debriefing^28^ and to revisit and discuss themes. Analysis of 32 interviews indicated that data saturation had been reached for both codes and meaning.^29^ Themes are presented in this manuscript using text descriptions and illustrative quotations that were selected from transcripts of interviews. All study investigators reviewed and agreed on final themes, subthemes, and illustrative quotations.

Demographic characteristics were also collected during interviews, organized in Microsoft Excel, and summarized by medians (interquartile ranges) or by number and percent.

### Ethical Approval

Ethical permission to conduct this study was obtained from the National Institute for Medical Research in Dar es Salaam, Tanzania (NIMR/HQ/R.8a/Vol.IX/2284) and from Weill Cornell Medicine in New York, United States (1604017171).

## RESULTS

During August 2021, our team conducted 32 in-depth interviews with those identified as Muslim leaders, which lasted approximately 30–45 minutes each. Among those interviewed, 17 were male and 15 were female. The median participant age was 39 years (interquartile range: 28–47), and the majority (82%) were married. Participants reported having a median of 4 (interquartile range: 2–6) children. All male participants were considered official religious leaders in their communities, while all women participants were lay leaders. Additional details of study participants are provided in Table 1.

**TABLE 1.** Demographic Characteristics of Muslim Religious Leader Study Participants in Rural Tanzania

**Characteristics**	**Total** **(N=32)**	**Female** **(n=15)**	**Male** **(n=17)**
Age in years, median (IQR)	39 (28–47)	33 (28–45)	43 (29–61)
Number of children, median (IQR)	4 (2–6)	3 (2–4)	5 (2–10)
Married, No. (%)	27 (84)	13 (87)	14 (82)
Converted to Islam in adulthood, No. (%)	4 (13)	4 (27)	0

Abbreviation: IQR, interquartile range.

Overall, interviews revealed divergent views among religious leaders regarding contraceptive use and its acceptability for followers of Islamic faith. We found a strong consensus that Islam promotes birth spacing and that breastfeeding, the calendar method, and the withdrawal method are generally viewed as acceptable methods to plan one’s family and space births. Muslim leaders consistently stated that Muslim children have a right to be raised with good health and that both birth spacing and breastfeeding help ensure this. Attitudes and trust toward doctors and modern contraceptives were more varied.

### Islam and FP

#### Qur’an Supports Birth Spacing to Protect the Health of Infants

All 32 leaders affirmed that Islam encourages birth spacing. Specifically, both men and women stated that Allah said in the Qur’an that there should be 2 years between births for a healthy child to be born.

*There is evidence in the Qur’an that family planning exists and it is there to protect the human interest.* —Man, aged 24 years

All leaders interviewed affirmed that Islam encourages birth spacing.

Safeguarding the health and well-being of the child was the prevailing reason that participants thought the Qur’an promotes birth spacing. Several cited examples of Muslim role models, including Hadija, the first wife of Muhammad, whose children were believed to be healthy because she was using FP to give birth. Others explained how adherence to this directive exemplifies faithfulness to Islamic family values.

*What I know about family planning, my religion teaches me to have children with an interval.* —Man, aged 46 years

Several participants mentioned that spacing births helps to ensure that children will follow parents’ Islamic faith because parents would have time to educate their children properly.

*The Qur’an says that family planning exists. The Qur’an is leading you to have few children so that you can teach them religious values.* —Man, aged 45 years

*In the past when we married, you see we even had 10 children because we did not have family planning education. Life is hard if you give birth to 10 children, you cannot raise them well.* —Man, aged 83 years

Some leaders noted that not all community members believed that the Qur’an supported FP. One leader described challenges in educating community members because of questions around the belief that Allah has created people to have children. However, this participant, like most others in our study, believed that Allah has said there should be a plan for births, including breastfeeding for 2–3 years before having another child, and urged that further community education was needed to encourage FP.

#### Generally Accepted Ways to Space Births

Leaders generally agreed on several acceptable methods that can be used to space births. Breastfeeding was viewed as protecting a child’s essential right to health and thus was unanimously approved by both men and women as the first and foremost way by which FP occurs.

*You give birth to a child and then you are raising that child in the Islamic religion as we are taught that a child should be breastfed for 2 years. Then you should stop breastfeeding him so that when she gets another pregnancy then the other child will have the right of breastfeeding, so it’s to give a child a right of being born and raised.* —Man, aged 46 years

Breastfeeding was unanimously approved by both men and women as the first and foremost way by which FP occurs.

Islamic women also viewed breastfeeding for 2 years not only as a religious duty but as a child’s “basic right” to be born and belonging to the Islamic faith. Though several participants referred to the practice of breastfeeding to space births, none explicitly demonstrated an understanding that exclusive breastfeeding provides more effective pregnancy prevention than nonexclusive breastfeeding.

Two additional well-accepted methods of FP included withdrawal during intercourse and the calendar method, which tracks the length of the menstrual cycle to avoid intercourse during a woman’s most fertile days. No participants specifically mentioned that the effectiveness of breastfeeding as a form of contraception wanes after 6 months. However, they did indicate the use of other methods in conjunction with breastfeeding for 2 years.

*The presence of breastfeeding for 2 years means that it is the use of family planning and among the family planning methods are calendar method, using condoms, and withdrawing so that you can protect the child’s life.* —Man, aged 28 years

Leaders who endorsed the withdrawal and calendar methods often described having successfully used these methods themselves. Several men mentioned that Muhammad did not forbid the use of withdrawal when speaking with his companions, which is described in Sahih Muslim Book 8, Hadith Number 3388. Similarly, others explained that they understood the calendar method to have been introduced by him.

*Prophet Muhammad (SAW) has explained to us that when a woman is in her dangerous days [around the time of ovulation] you are not allowed to seduce her. Also, when she is breastfeeding we are supposed to stay away from them and take care of them well with so much love but we have to wait to have sexual intercourse with them until they have finished their dangerous days so that they can breastfeed the child.* —Man, aged 46 years

Female leaders also generally approved the calendar method and asserted that “Islamic family planning is the best” and that this takes the form of a calendar method. Another participant explained that she teaches other women about the calendar method.

*She should plan her family, as she enters her menstrual cycle, she should know when to have sex with a man.* —Woman, aged 39 years

### Islam, Modern Contraceptives, and Doctors

#### More Debated Ways to Space Births

In contrast, modern contraceptives such as condoms, birth control pills, implants, intrauterine devices, and injections were viewed as less acceptable by many Muslim leaders. Some stated that condoms are strictly forbidden. However, others listed condoms, withdrawal, and the calendar method as acceptable FP methods. One leader who was in favor of withdrawal and the calendar method stated how he had advised mosque members.

*Understand that [when] she is in her ovulation days that it is dangerous to have sexual intercourse, or else to use a condom, which is a right method.* —Man, aged 28 years

Another leader explained his nuanced view that breastfeeding is best but condoms are acceptable.

*[It is] the method we know in our religion, [but] the second [best] method is the method of protecting yourself, for example you can use these modern methods like condoms.* —Man, aged 52 years

Many leaders grouped oral contraceptive pills, implants, intrauterine devices, and injections together, sometimes referring to these as “government” or “professional” methods. Among those who considered these methods unacceptable, 2 key objections emerged. First, many leaders feared that these contraceptives were dangerous to women’s health and capable of causing disease, infertility, and cancer. Given their view that any substance that could harm the body is prohibited by Islamic law, they stated that these contraceptives cannot be used.

*We are told in the Qur’an to use family planning methods that have no side effects.* —Woman, aged 30 years

The second major objection was that the absence of modern contraceptives in the Qur’an was interpreted as prohibition.

*The prophet did not teach about implants because he said that those things bring effects and they should not use methods which will affect them.* —Male leader, aged 29 years

Others supported using these modern contraceptives, and 2 women leaders aged 20–29 years admitted to using implants themselves. One male leader reported that the most accepted method in his community is contraceptive pills. Another male leader espoused the positive results of the use of implants and pills in his community. One man stated that he preferred pills because following the calendar method requires being attentive for adherence.

*[The] situation of should I use these other things or should I be looking at my wife’s calendar every time to see when she gets pregnant… becomes difficult and the direct solution is to use pills.* —Man, aged 24 years

Leaders who accepted modern contraception recognized the controversy around these methods in Islam. A leader who herself was using a contraceptive implant stated that some in her religious community were opposed to modern methods.

*[They] consider family planning as blasphemy [because] God said that we should reproduce.* —Woman, aged 27 years

Similarly, a female leader spoke positively of modern contraceptive use in her community.

*They mostly use injections and implants; I hear about them a lot… positive impacts have been obtained, [but some members] consider it as something bad which may cause problems, so there are those who agree with it and those who don’t.* —Woman, aged 66 years

The source of some of this tension was summarized in one leader’s statement.

*The use of family planning is right according to Holy Books like the Qur’an, but God tells us to give birth and multiply on earth.* —Man, aged 28 years

#### Perspectives on Modern Medicine

Of the 32 Muslim leaders interviewed, 10 expressed mistrust of modern medicine and sometimes of science and did not accept FP methods recommended by doctors.

*In the Islamic religion it is wrong to use the scientific family planning methods and most of them [health professionals] will just use the scientific methods.* —Male, aged 61 years

One participant further expanded this sentiment toward suspicion of modern education in general, exemplifying that this mistrust of modern medicine and contraceptives may be rooted in a deeper mistrust of external, nonlocal systems that could create unwanted change in the society overall and in methods of FP.

*A person comes and takes a youth so that he can take him to school, he goes and learns nullified things and later comes back to the village… starts his own mosque and misleads the mosque members that, do not do this and do not do this.* —Female, aged 26 years

Importantly, most of the leaders who expressed mistrust of modern medicine did support birth spacing and wanted their communities to learn about FP. Compared to Muslim leaders who expressed more trust in modern medicine, these leaders insisted that teaching about FP should come from leaders, like sheikhs and imams, to maintain faithfulness to Muslim principles.

*Our sheikhs teach us about safe family planning, the one that is allowed in our religion and unluckily, there are other family planning methods that are being brought here by the professionals which we see them, they are not safe.* —Male leader, aged 63 years

Most of the leaders who expressed mistrust of modern medicine did support birth spacing and wanted their communities to learn about FP.

By contrast, other leaders expressed acceptance of modern medicine and tended to view contraceptives, such as intrauterine devices, injections, and implants, as acceptable and efficient. Some described their own positive experiences with the medical system. A woman who had developed a tumor and had been hospitalized for treatment reported that, after taking prescribed medications, she was able to have a child who is now aged 6 years. Leaders who accepted these contraceptives expressed a need for the assistance of health professionals to teach both them and their communities about FP. Many reported that their communities had poor knowledge of FP that they anticipated would persist without education from a specialist.

Interestingly, some comments from religious leaders who supported only breastfeeding and/or the calendar method indicated respect for health providers’ views and possibilities to build bridges between the 2 groups. For example, a male leader bolstered his views by noting that even health professionals recommend breastfeeding for 2 years and then additionally mentioned condoms and oral medications as other methods of which he was aware. Another man, when asked about FP methods, responded:

*There is calendar method which is being explained by scientists [and is] the safest family planning method to use.*

Notably, this participant was vehemently opposed to many modern contraceptives.

*Methods that are being given by these scientific doctors, they have major challenges [such as oral contraceptives leading to] women not getting their menstrual periods and it results in cervical cancer.* —Man, aged 63 years

### Important Aspects for Implementing FP Education Among Muslims

All leaders responded positively when asked their opinion regarding a seminar that would provide education about FP among their religious communities, affirming that education for community members was deeply needed. Several leaders urged the interviewers to come back and help mobilize the community, and others strongly advised the study team to return to support such a seminar.

All leaders stated that they would be willing to teach such a seminar, and several recommended that Muslim religious leaders would be the most appropriate to teach about FP.

*We should use the systems with religious foundation. Sheikhs, pastors, priests, bishops must come so that they can be taught how to teach family planning. Then after that, the community can be given education… when you use religious teachings, to be honest, you will be able to accomplish the family planning program because there is no religion that says that people should just give birth with no plan.* —Male, aged 29 years

Another leader also noted the high influence of Muslim leaders to address this issue.

*I think they should be educated in the mosque and religious leaders are the ones to be educated more because they are the ones who are supposed to be close to the mosque members. When they influence them that on a certain day there will be a meeting, they will attend in a big number.* —Male, aged 52 years

Leaders were careful to point out that they would be comfortable teaching the FP methods that they themselves saw as acceptable. Some noted that they would feel comfortable teaching about methods that don’t have side effects for women or children. Another who wanted to teach about modern contraception volunteered to bring 2–3 people from his staff for training, noting that they needed professionals to teach them if they are to educate their community.

Leaders also offered practical suggestions for having the greatest community impact (Table 2). Most recommended that education about FP should take place on a Friday in the mosque, and some mentioned that it should occur during Ramadan when mosque attendance is highest. Others suggested distributing invitations in advance.

**TABLE 2.** Leaders’ Recommendations for Providing Teaching About Family Planning to Muslim Religious Leaders in Rural Tanzania

**Implementation Recommendation**	**Total Leaders, No. (%)** **(N=32)**	**Female Leaders, No. (%)** **(n=15)**	**Male Leaders, No. (%)** **(n=17)**
Recommendations for who should lead teaching sessions			
Muslim leaders	32 (100)	15 (100)	17 (100)
Medical professionals	5 (16)	1 (7)	4 (24)
Recommendations for where, when, and how to convene teaching			
Not in the mosque^a^	4 (13)	2 (13)	2 (12)
In the mosque with specific mention of Fridays	20 (63)	11 (73)	9 (53)
In the mosque with specific mention of Ramadan	3 (9)	0 (0)	3 (18)
Formal invitations for participants	6 (19)	2 (13)	4 (24)
Recommendations for teaching men and women			
Careful attention to men’s and women’s seating	32 (100)	15 (100)	17 (100)
Men and women can be put together for the seminar	12 (38)	8 (53)	4 (24)
Men and women can sit in the same room but apart for the seminar	7 (22)	3 (20)	4 (24)
Men and women cannot/should not be educated together (location not relevant)	4 (13)	3 (20)	1 (6)
Women will not be comfortable speaking in front of men	4 (13)	3 (20)	1 (6)

^a^ Teaching not in the mosque was particularly recommended by people who emphasized teaching men and women together.

Leaders presented divergent views on whether men and women should be taught together versus apart. Some religious leaders strongly favored a single seminar taking place for both men and women.

*[This will allow] everyone [to be] explained the effects and the benefits of family planning if it will be prepared with the required values.* —Man, aged 63 years

*It will be great because all of us will understand.* —Man, aged 82 years

A female leader said the sheikh would have authority to bring men and women together.

*As the sheikh is the one responsible to speak with the believers in the mosque so as to be able to bring together women and men and sit together.* —Woman, aged 48 years

Some leaders stated that men and women could be in the same room for the seminar but with men on 1 side and women on the other side, while others thought that distinct seminars should take place for men and for women. These stark variations suggest the optimal and most culturally appropriate ways to reach men and women may vary in different communities and should be determined by the leaders of that community.

The optimal and most culturally appropriate ways to reach men and women may vary in different communities and should be determined by the leaders of that community.

Several leaders broached the question of women being able to speak during these seminars, with varying opinions. A male leader struggled with the community norm being that women cannot speak in services.

*Because it is an education provision, it will be hard to use the same system of women not talking but to write what they want to say on the paper.* —Man, aged 24 years

Another male leader suggested that women should have separate sessions because women would not feel comfortable speaking and asking questions if they were together with men.

*[Women] are afraid to talk in the men’s group but she talks when she is with her fellow women.* —Man, aged 46 years

This sentiment was similarly expressed by a female leader.

*If we talk about it in the mosque, it’ll be a bit hard due to the presence of men, [but in a woman’s only meeting] you may talk about everything that is required without hesitation with your fellow woman, as long as you are isolated from men.* —Woman, aged 66 years

These comments suggest that, at the very least, breakout sessions separating men and women are a critical component of facilitating discussions and clarifications surrounding FP.

## DISCUSSION

We report that spacing births is viewed as desirable and concordant with Islamic beliefs, despite a diversity of views on modern contraceptive methods. Moreover, Muslim religious leaders unanimously expressed a willingness to learn more about FP strategies and to educate their communities. Our data support previous findings from Kenya, Malawi, and Zimbabwe, not only by illustrating the diversity of these views within the Islamic faith but also by indicating a potentially effective way forward for increasing faith communities’ understanding of FP.^20^^,^^26^^,^^30^ Additionally, the United Nations Population Fund-Tanzania has worked with Muslim religious leaders in Zanzibar, where a large majority of the population is Muslim, to encourage modern contraceptive use, further demonstrating the potential effectiveness of this strategy.^31^^,^^32^ Drawing on Muslim religious leaders’ esteem for some effective strategies like breastfeeding and the calendar method, as well as providing accurate information about the potential harms presented by both the use of contraceptive methods and failing to space births, we posit that our data offer a novel way forward for promoting healthy planning of families in Muslim communities. Addressing gaps in knowledge and ability to space births within Muslim communities is of critical importance in sub-Saharan Africa, where more than 15% of the 1.14 billion inhabitants identify as Muslim.^33^

Our findings highlight the fundamental importance of working with trusted community leaders to understand how Islamic beliefs impact FP and how to develop effective FP educational interventions. The Muslim leaders interviewed in this study all perceived that Islamic law encourages spacing children to provide children with their fundamental rights, ensure their health, and raise them in the Islamic faith.^25^^,^^34^^–^^36^ Further, almost all leaders stated that their communities needed more education about FP and that they themselves would be motivated to provide this education. Many leaders reported having observed a need for birth spacing and having already discussed this topic with some members of their communities.

Our findings highlight the fundamental importance of working with trusted community leaders to understand how Islamic beliefs impact FP and how to develop effective FP educational interventions.

Amid diverse perceptions about contraceptives, our data reveal some areas of concordance between Muslim leaders’ beliefs and the efficacy of certain contraceptive methods. For example, all participants agreed that breastfeeding was an appropriate way to practice birth spacing that was endorsed by the Qur’an. Exclusive breastfeeding effectively prevents all except 2 in 100 pregnancies for the first 6 months of a newborn’s life, but after 6 months, effectiveness decreases sharply.^37^ No religious leader in our study directly mentioned the decreasing effectiveness of breastfeeding to prevent pregnancy after 6 months, but many acknowledged the use of other methods concurrently, particularly the calendar method. The overall acceptance of both breastfeeding and the calendar method as methods of birth spacing suggests that an educational seminar providing clear, accurate teaching on efficacy and correct implementation of these methods would be well received. Comparative efficacy of exclusive versus nonexclusive breastfeeding should also be emphasized.

The Standard Days Method, a rigorous counting system using beads that provides contraceptive efficacy of 95% when used perfectly and ∼88% when used more typically,^38^^–^^40^ has been implemented in some low- and middle-income countries with good success but has not been broadly implemented in Tanzania.^40^^,^^41^ Our preliminary data suggest that the Standard Days Method would be widely acceptable and could improve birth spacing in Muslim communities.

The mistrust of physicians and modern contraception, described by several leaders, seems fueled both by the Islamic teaching that people must not use substances that harm the body^42^^–^^47^ and by misconceptions about the safety of modern contraceptives. We and others in many sub-Saharan African countries have previously documented widespread views that contraceptives can cause cancer, infertility, and birth defects.^17^^,^^18^^,^^48^^,^^49^ The challenge of these views is that while they hold some truth (e.g., slightly increased risk of cervical cancer in those using oral contraceptives longer than 5 years),^50^ they do not incorporate the reality that short interpregnancy intervals can also increase risks of infant mortality, preterm birth, and, in women aged over 35 years, maternal mortality.^2^^,^^51^^,^^52^ Based on our experience addressing many of these same concerns about modern contraceptives among Christian communities, we anticipate that leaders will benefit from and appreciate receiving simple, scientifically accurate information about risks and benefits. Dissemination of this information should include both the rates and types of harm associated with using various modern contraceptives and the possibility of harm from closely spaced births when modern contraception is not used. Fostering partnerships between medical professionals and trusted Muslim leaders will be essential for delivering FP information to community members that will be accepted. Additionally, we plan to invite some of the leaders who were interviewed for this study to serve as liaisons and co-educators during the education sessions. Because the seminar will take place in small groups, we aim to implement the teachings in a way that is conversational and allows leaders to express their opinions and ask questions.

Our data provide a way forward for designing an educational FP seminar for Muslim religious leaders that can engage and empower them to discuss FP in their own communities. For a seminar with a small number of Muslim leaders, our findings suggest that men and women can learn together if the education is provided in a nonreligious setting. Previous data show that men prefer education from educators who are men and that women prefer education from women,^53^ so this religious leaders’ seminar would include both male and female educators and would be co-taught by Muslim religious leaders and Muslim physicians. Our data also highlight that, after a joint educational session, male and female religious leaders will additionally benefit from separate discussions with educators of the same sex, allowing them to ask questions more freely.

Our data provide a way forward for the design of an educational FP seminar for Muslim religious leaders that can engage and empower them to discuss FP in their own communities.

The diversity of perspectives represented in our interviews with Muslim leaders, as well as in a small study in southeastern Tanzania,^54^ indicates that these leaders’ subsequent teaching about FP in their communities needs to be highly context specific. Our goal would be to provide knowledge and tools to equip these leaders to bring accurate FP information to their communities in the way they believe would be most appropriate and effective. When providing education to religious leaders, we would highlight considerations of respecting religious traditions, balanced with the benefits of men and women receiving the same teachings. The goal of our educational seminar would be to provide Muslim leaders with knowledge about both traditional methods of FP and modern contraceptive methods, including how to use these methods correctly, efficacy rates, and risks and benefits. The seminar would prioritize Muslim leaders’ understanding of the methods that are widely practiced, including explaining how the efficacy of breastfeeding as a contraception method decreases after 6 months and is completely ineffective at 12 months and the most effective ways to implement the calendar method. Together, leaders would be able to brainstorm strategies to bring the appropriate information to their communities, and ultimately, they would decide which methods to teach and how to deliver these teachings in their communities.

### Limitations

Our study, like most qualitative studies, is likely to be limited by social desirability bias. Because participants knew the interviewers came from the city of Mwanza, participants may have felt less free to share views that they may have feared that interviewers could perceive as rural or uneducated. Questions were open ended, and data saturation was reached for both men and women, suggesting that most people did freely share their true opinions. Certain contextual aspects of FP, such as views that having few children could indicate trials and tribulations within marriage and that having more children is equated with greater possessions,^24^ were not mentioned in our interviews, lending evidence to the possibility of omission during interviews. However, participants were open regarding other topics, including disclosing their opinions on different contraceptives and, in some cases, sharing their own positive or negative experiences with these methods. We did not specifically ask about personal experiences with FP, though many participants shared these. Although data were also collected from only 1 region in northwest Tanzania, the villages in which we worked are spread over a large distance and relatively isolated from one another, and views expressed were widespread. This suggests the generalizability of our findings in rural areas beyond those studied.

## CONCLUSION

In summary, we have identified opportunities to work with Muslim leaders to promote birth spacing and enhance understanding of FP in ways that are concordant with Islamic teaching. Data derived from these interviews will be used to design and pilot-test an educational intervention for Muslim religious and lay leaders. After the intervention, we will collect additional qualitative data from both male and female trusted community leaders to refine this intervention for broader implementation and continue striving to improve maternal and child health in sub-Saharan Africa.

## Supplementary Material

GHSP-D-22-00204-supplement.pdf

## Notes

**Peer Reviewed**

**First published online:** January 20, 2023.
