# Supplementary material for: Perspectives of Muslim Religious Leaders to Shape an Educational Intervention About Family Planning in Rural Tanzania: A Qualitative Study
Source: Glob Health Sci Pract. 2023 Feb 28;11(1):e2200204. doi: 10.9745/GHSP-D-22-00204 (PMC9972385; doi:10.9745/GHSP-D-22-00204)
Supplement: GHSP-D-22-00204-supplement.pdf [file GHSP-D-22-00204-supplement.pdf]

Supplement to: Chalem A, Nzali A, Cordeiro A, et al. Perspectives of Muslim religious leaders to shape an educational intervention about family planning in rural Tanzania: a qualitative study. *Glob Health Sci Pract*. 2023;11(1):e2200204. https://doi.org/10.9745/GHSP-D-22-00204

Interview Guide for Muslim Leaders

1. How old are you?
2. Are you married?
3. How many children do you have?
4. What do you know about family planning?
    a. What methods of family planning are you familiar with or do you know about?
5. In your opinion, do you think many people in your community know and understand what family planning is?
6. As a religious leader who is responsible of fostering the growth of your people. In your experience of giving advice to various people, have you ever advised anyone about family planning?
    a. Have you ever faced any challenges concerning family planning?
    b. Do you think the advice you offered was beneficial or effective?
7. In your expert opinion, what do you think the Quran says about the use of family planning among Muslims?
    a. If so, could you provide us with verses from the Quran that talk about family planning?
8. Some people use Prophet Muhammad's (s.a.w) Hadiths to either support or challenge the use of family planning. How do you think Prophet Muhammad's (s.a.w) Hadiths talk about family planning?
    a. Are there any Hadiths that you can think of that indirectly or directly talk about family planning?
9. You are both a community member and a religious leader within your community, do you think that your community needs more education regarding family planning?
    a. What would be the benefit/s of family planning?
10. What forms of family planning do you think would be most acceptable within your community?
    a. Do you think they have brought about positive results/changes within your community?
11. Do you think it would be appropriate if a religious leader teaches about family planning in the mosque?
    a. What time do you think would be more appropriate to provide this education?
12. Would you feel comfortable teaching about family planning, as a religious leader within the community, if you were to first receive training to talk about it from a health and Islamic perspective?
13. Do you think women have a different opinion of family planning than men within the community?
    a. If so, how are women's opinions different?
14. We are in process of preparing a seminar about family planning. What structure would you recommend for us to use in order to target and incorporate both women and men?
15. What Muslim denomination are you?
16. How many worshippers attend this mosque?
